# Effects of single-modification/cross-modification of starch on the mechanical properties of new biodegradable composites

**DOI:** 10.1039/c8ra01592a

**Published:** 2018-04-03

**Authors:** Shuai Chen, Fang-yi Li, Jian-feng Li, Xu Sun, Jin-feng Cui, Chuan-wei Zhang, Li-ming Wang, Qi Xie, Jie Xu

**Affiliations:** Key Laboratory of High Efficiency and Clean Mechanical Manufacture (Ministry of Education), School of Mechanical Engineering, Shandong University Jinan 250061 China; National Demonstration Center for Experimental Mechanical Engineering Education, Shandong University Jinan 250061 China

## Abstract

Starch-based composites with different modified starches were prepared by combining starches with sisal fibers to investigate the effects of single-modification/cross-modification of starch on the mechanical properties of new biodegradable composites. Mechanical test results showed that cross-modification of starch improved the toughness of the composites, whereas single-modification improved the tensile strength. The oxidized esterified starch-based composite (OESC) exhibited the best toughness, with improved elongation at break and Young’s modulus by 136.1% and 54.3%, respectively, compared with a native starch-based composite. Meanwhile, the tensile strength of the esterified starch-based composite (ESC) improved by 61.6%. The hydrogen bonds, crystallinity, and micro-structure of the composites were investigated to reveal the inherent mechanism of the changes in performance. Fourier transform infrared spectroscopy showed that modification of starch changed the functional groups of starch. Thus, the ESC formed the strongest hydrogen bonds. X-ray diffraction analysis showed that the crystallinity decreased after the starches were modified. The OESC exhibited the lowest crystallinity, with a severely damaged structure. Many starch branches were combined with sisal fibers so that the composite was not easily pulled off. Scanning electron microscopy images showed that the OESC formed good cell structures internally when starch uniformly attached to the surface of the fibers.

## Abstract 2

Starch-based composites with different modified starches were prepared by combining starches with sisal fibers to investigate the effects of single-modification/cross-modification of starch on the mechanical properties of new biodegradable composites.

## Graphical abstract

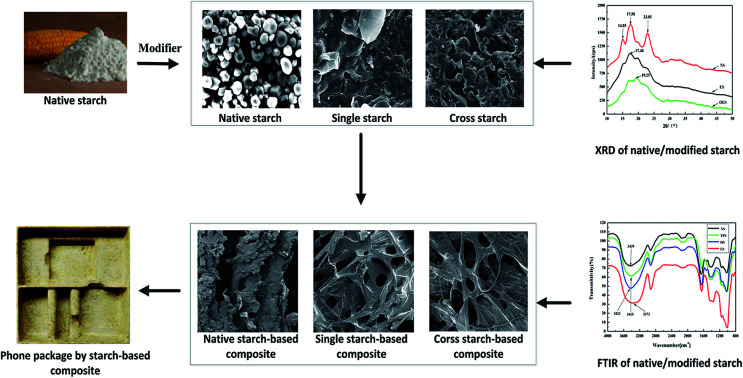

## 1. Introduction

New biodegradable composites are a type of starch-based composite, a kind of material with plant fiber as the skeleton and starch as a binder.^1–3^ Fiber and starch can not only effectively alleviate the problem of “white pollution”, but also make full use of biological resources because of the advantages from using renewable raw materials from a wide range of sources which are biodegradable. Therefore, starch-based composites have become a research ‘hot spot’ worldwide.^4^

Numerous scholars have conducted multiple studies on biodegradable starch-based composites.^5,6^ Mir *et al.*^7–9^ studied starch-based composites reinforced by different types of plant fibers. Mechanical property test results showed that the mechanical properties of the composites were best when reinforced by sisal fibers. The micro-mechanism which gave rise to the differences in mechanical properties was found by microscopic analysis to be that the combination of sisal fibers and starch was so strong that the tensile strength was improved. Yusoff *et al.*^10–12^ found that native starch (NS) was bonded tightly by a series of colloidal molecules to form a relatively closed internal structure. The NS crystallinity was extremely high, hindering its combination with other polymers. These factors led to the poor mechanical properties of the NS-based composite. Guimarães *et al.*^13,14^ prepared starch-based composites from plant fibers. The tensile test results showed that starch modification and the mechanical strength of the composites were closely related. The reason was that the degree of crystallinity of the modified starches was reduced, leading to the change in the internal structure of the starches. Thus, despite the poor mechanical properties of NS-based composites, the problems can be solved by modifying the starch. Starch modification is an effective solution, thus, studies on modified starch are valuable.

To date, starch modifications include plasticization,^15,16^ oxidation,^17^ esterification,^18^ etherification,^19^ graft copolymerization,^20^ and cross-modification.^21^ Angellier *et al.*^10,22,23^ studied the mechanism of the plasticization modification of starch and found that plasticizers are typically low-molecular-weight substances that can be easily incorporated into the polymer matrix. This can destroy the molecular structure of starch and lead it to form extremely strong hydrogen bonds. The mechanical properties of starch-based composites have been significantly improved. Compound plasticizers, which are composed of glycerol and ethylene glycol, exert good plasticizing effects. Moreover, the best plasticization ratio was *m*_starch_ : *m*_plasticizer_ = 10 : 3. Zhang *et al.*^24,25^ explored the best process for preparing oxidized starch and produced oxidized starch with a high degree of substitution. Mechanical test results showed a considerably enhanced mechanical performance of an oxidized starch-based composite compared with the NS-based composite. H_2_O_2_ is the most suitable oxidizer, and the optimal oxidation ratio is *m*_starch_ : *m*_oxidant_ = 10 : 1.5. Z. F. Wang *et al.*^26,27^ prepared esterified starch (ES) with good ductility, viscosity stability, good film formation and high transparency. Starch/natural rubber composites were prepared by mixing ES and the natural rubber latex. The results revealed improved thermal stability and mechanical properties, especially for the tensile strength of these composites compared with NS. In addition, the corn starch after esterification had significantly reduced moisture sensitivity and surface hydrophilicity. The team concluded that the esterification modification gives an excellent synthesized performance compared with other single-modification starches. Polylactic acid/etherified starch composites were prepared by Liu *et al.*,^28–30^ and the thermal stability and the tensile strength of etherified starch-based composites presented a similar trend in mechanical properties as ES. However, other aspects of the performance were obviously inferior to ES. Hebeish *et al.*^31–33^ optimized the synthesis of graft-copolymerized starch and applied the modification. The results showed that modified starch exhibits good rheology. Zhang *et al.*^34^ studied the effects of plasticization and oxidation on starch-based composites. Their findings revealed that cross-modified starch-based composites display advantages in some aspects, such as cushioning properties and water resistance, *etc.* However, the effects of single-modification and cross-modification of starch on the properties of composites were not investigated in detail in his study. A relatively uniform open cell structure was formed in the plasticized oxygen-based composite with certain cushioning properties. The starch-based composites had widely distributed cell structures. Due to their sound-absorbing, heat-insulating, and cushioning properties, starch-based composites can be used in different applications, including transportation, packaging, and interior decoration.^35,36^ In summary, we can draw the following conclusions. (1) Starch-based composites reinforced by sisal fibers exhibited the best mechanical properties. (2) For the purpose of this study, ES showed better properties and a higher research value compared with other single-modification starches. (3) Cross-modification starches presented superior properties compared with single-modification starches in some aspects. To date, although scholars have found that the single-modification/cross-modification of starch exerts different effects on the performance of composites, no systematic study can prove this phenomenon and the corresponding microcosmic mechanism. It is very meaningful that we can demonstrate this phenomenon by conducting experiments as it will have a direct effect on the manufacture of cushioned packaging.

In this study, extensive research on the above problem has been carried out to demonstrate that we can modify starch according to our requirements to prepare products with specific performances. Single-modified/cross-modified starch-based composites were prepared at the optimal modified ratio in accordance with the aforementioned ratio by molding foam at a certain temperature. Only the ES was further modified in this article owing to its high research value. The single-modification starches included plasticized starch (TPS), oxidized starch (OS), and esterified starch (ES). The cross-modification starches included plasticized esterified starch (TPES) and oxidized esterified starch (OES).

Through tensile and compression tests, the tensile curves and cushioning coefficient curves of the composites were obtained. Toughness was characterized by the elongation at break and Young’s modulus in this paper. A large elongation at break and a small Young’s modulus meant that the composites had a high toughness. The microscopic mechanism for the difference between the single-modification starch and the cross-modification starch was analyzed from the perspective of hydrogen bonds and crystallinity through Fourier transform infrared (FTIR) spectroscopy and X-ray diffraction (XRD), respectively. Scanning electron microscopy (SEM) showed that the starch and fiber were closely combined in the single-modified/cross-modified starch-based composites. The composites all formed a cell structure with different evenness and quantities, thus verifying the accuracy of the FTIR spectroscopy and XRD.

## 2. Experimental

### 2.1 Materials

Sisal fibers with an average length of 6–10 mm were self-made. The ratio of length to diameter of sisal fibers was 120. The binder, that is, corn starch with an average particle diameter of 80 nm, was purchased from Hebei Huachen Starch Sugar Co. Ltd. Glycerol and ethylene glycol (99% purity) as plasticizers were purchased from Tianjin Fuyu Fine Chemical Co. Ltd. H_2_O_2_ (99.5% analytical level) and esterified acetic anhydride (99% analytical level) were purchased from Luqiang Chemical Reagent Field in Jinan. NaOH, the foaming agent AR, and other additives were purchased from Yantai Shuangshuang Chemical Co. Ltd.

### 2.2 Preparation of modified starch

TPS was prepared according to the following procedures: NS (50 g) was mixed with distilled water (200 mL) in a round-bottomed flask. The flask was heated at 85 °C in a water bath for half an hour with mild stirring. Glycerol and ethylene glycol as plasticizers were added into the mixture with the best ratio (glycerol and ethylene glycol) of 2 : 1. The slurry was stirred at 120 rpm for 2 h in a constant-temperature water bath at 85 °C for 12 h.

OS was prepared as follows: distilled water (200 mL) and NS (50 g) were placed into a 500 mL round-bottomed flask in a constant-temperature water bath at 85 °C for 30 min with mild stirring. Then, 7.5 mL of the oxidant H_2_O_2_ and 5 g of the catalyst CuSO_4_ were added to the mixture with 20 mL of distilled water. The solution was added in drops into the gelatinized suspension after the temperature of water bath cooled to 25 °C. During oxidation, vigorous stirring was performed with a mechanical stirrer to ensure uniform dispersion of H_2_O_2_ into the gelatinized starch.

ES was prepared as follows: NS (50 g) was mixed with distilled water (200 mL) in a round-bottomed flask. The flask was heated at 85 °C in a water bath for 30 min with mild stirring. Esterified acetic anhydride (10 mL) was added to the mixture. The slurry was stirred at 120 rpm for 2 h in a constant-temperature water bath at 85 °C for 12 h.

TPES was modified through changing the plasticizer and esterifying agent. The preparation procedure was similar to that for TPS formulation, but ES was used instead of NS as in the preparation of TPS.

OES was modified through changing the oxidant and esterifying agent. The preparation procedure was similar to that for OS formulation, but ES was used instead of NS as in the preparation of OS.

Lastly, all mixtures were subsequently washed 10 times with 250 mL of distilled water.

### 2.3 Preparation and molding of starch-based composites

**Fig. 1:**
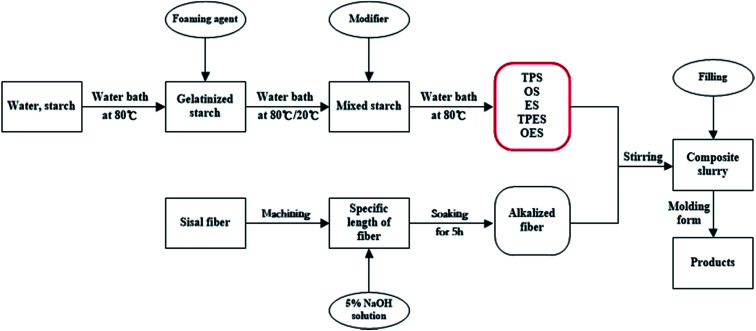
Flow chart of the preparation of starch-based composites.

Fig. 1 shows the preparation of composites. The modified starches (including NS) and sisal fibers were mixed according to the mass fraction of 5 : 3.^37^ The mixture was placed in a mixing machine and strongly stirred for 30 min to obtain six groups of starch-based composite slurries. Then, the composite slurries were wrapped with plastic wraps and marked corresponding to the type of starch. The labels were NS composite, TPS composite, OS composite, ES composite, TPES composite, and OES composite.

**Fig. 2:**
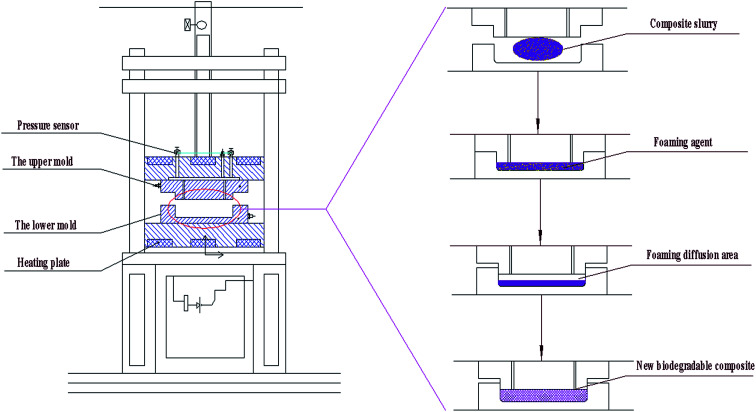
Flow diagram of the molding-foam of the new biomass-cushioned packaging products.

**Fig. 3:**
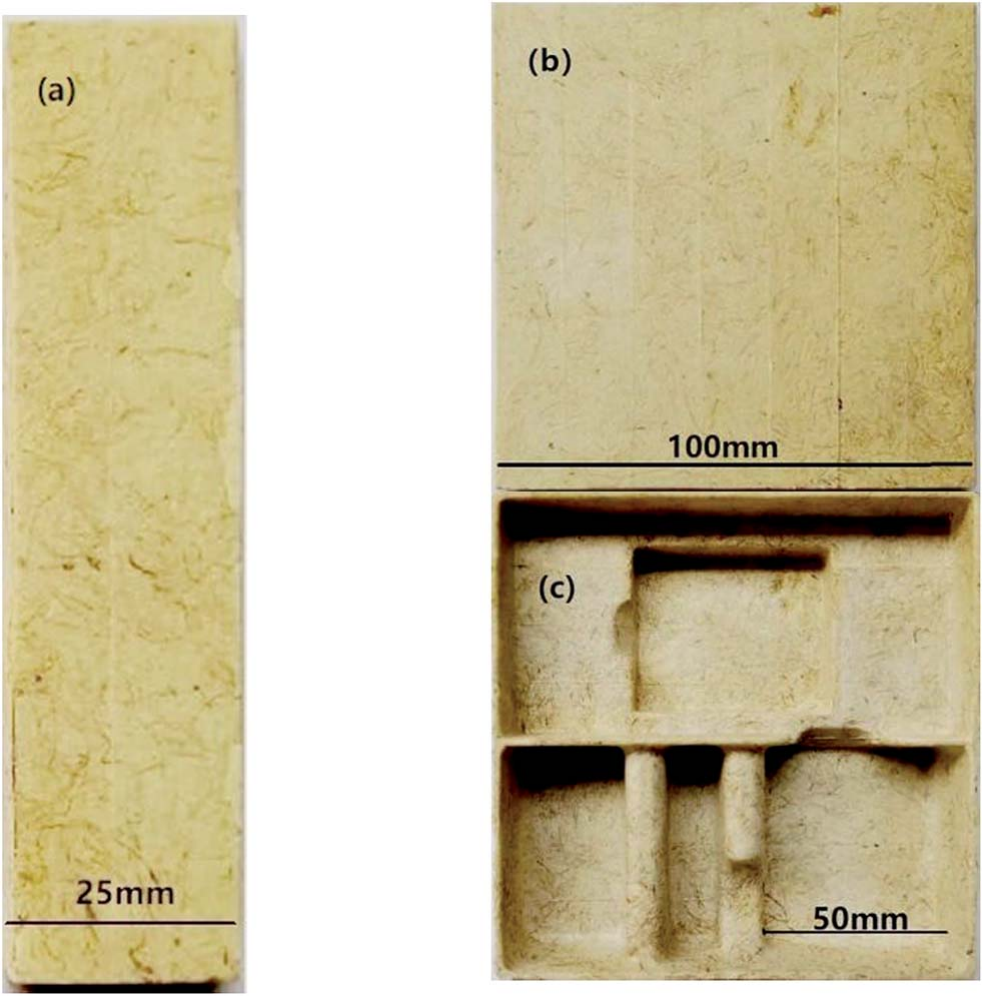
The new biomass-cushioned packaging products of (a) a tensile sample; (b) a compression sample; and (c) phone packaging.

Approximately 50 g of the six groups of composites were weighed and placed into a hot-pressing machine of the double column-single station as shown in Fig. 2. The temperature of the upper mold was set to 200 °C. The temperature of the lower mold was set to 200 °C. The mold pressure was set to 3 MPa. Then, the mold was pressed for 30 s and dried for 120 s. During the process, the foaming agent (AR) began to foam. Thus, the interior of the composite formed an open cellular honeycomb structure. Fig. 3(c) presents our phone packaging product and test samples with an open cellular honeycomb structure. In addition, other product shapes were prepared as needed.

### 2.4 Mechanical property testing

#### 2.4.1 Tensile strength test

Samples (100 mm × 25 mm × 5 mm)^38^ were tested using the standard method according to the national standard (Fig. 3(a)). Tensile specimens were drawn using a smart electronic tensile tester at 25 mm min^−1^ for tensile strength testing.

#### 2.4.2 Compression strength test

Samples (100 mm × 100 mm × 25 mm)^39^ were tested using the national standard method (Fig. 3(b)). The compressor exerted a force of 5 kN, and the platen increased the load along the thickness direction of the test specimen at a rate of 10 ± 2 mm min^−1^. The speed of the tester was set to 12 mm min^−1^ until the machine crushed the specimen. The thickness of the sample was taken as the original thickness.

All tests were performed using five samples, and the average of the data was obtained.

#### 2.4.3 Cushioning performance test

Test data were obtained by a compressive strength test, and the stress–strain curves of the samples were plotted to calculate the increment of the unit volume of the samples under different stress conditions to obtain the cushioning coefficients.^40^ Thus, a cushioning coefficient–stress curve (*C*–*δ* curve) was drawn.

The basic steps to determine the cushioning factor (*C*) and to plot the *C*–*δ* curve were as follows.

(1) The area under the stress–strain curve was divided into several small areas. A small divided area means high data accuracy.

(2) The values of each of *δ*_*i*_ and *ε*_*i*_ on the stress–strain curve (where *i* = 1, 2, 3, …) were recorded.

(3) The increment of strain energy for each stress section was determined, that is, each divided area was calculated.1
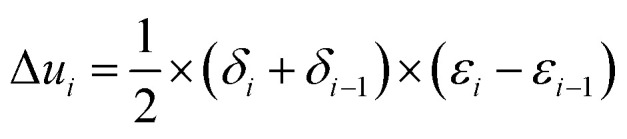


(4) Strain energy corresponding to each stress *δ*_*i*_(*u*_*i*_) was calculated.

2 *u*_*i*_ = ∑Δ*u*_*k*_

(5) The cushioning coefficient corresponding to each stress *δ*_*i*_ was calculated.3
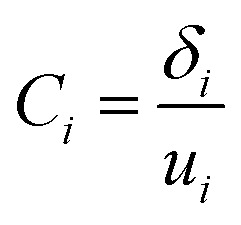


(6) The cushioning coefficient (*C*)–stress (*δ*) curve was plotted with the cushioning coefficient *C* as the ordinate and the stress *δ* as the abscissa.

### 2.5 Micro-characterization of modified starches and composites

#### 2.5.1 Infrared spectra analysis

Anhydrous NS, TPS, OS, ES, TPES, and OES of approximately 5 mg each were mixed with 150 mg KBr and milled thoroughly to reach a particle diameter of <2.5 μm. The mixtures were compressed into pellets under approximately 10–12 MPa and analyzed using a VERTEX-70 FTIR spectrometer. Spectra were recorded at a resolution of 2 cm^−1^ for 400–4000 cm^−1^.

#### 2.5.2 X-ray diffraction experiments

Starch samples were dried in a vacuum oven at 80 °C for 12 h. Anhydrous starch was ground in an agate mortar and passed through a 200 mesh screen. The sample was flattened to make the surface parallel to the glass frame. The assay was operated at room temperature using Ni-filtered Cu radiation and a curved graphite crystal monochromator. The slit system was DS/RS/SS = 1°/0.16 mm/1°. An angle (2*θ*) range of 5–65° was analyzed at a speed of 5° min^−1^.

#### 2.5.3 Scanning electron microscopy on composites

In starch-based composites, the spatial structure of composites was investigated using a scanning electron microscope (FEG-250) at an accelerating voltage of 10 kV. Prior to SEM, all samples were mounted on a piece of aluminium using a carbon ribbon and sputtered on the surface to result in a conductive sample.

## 3. Results and discussion

### 3.1 Mechanical property analysis of the composites

**Fig. 4:**
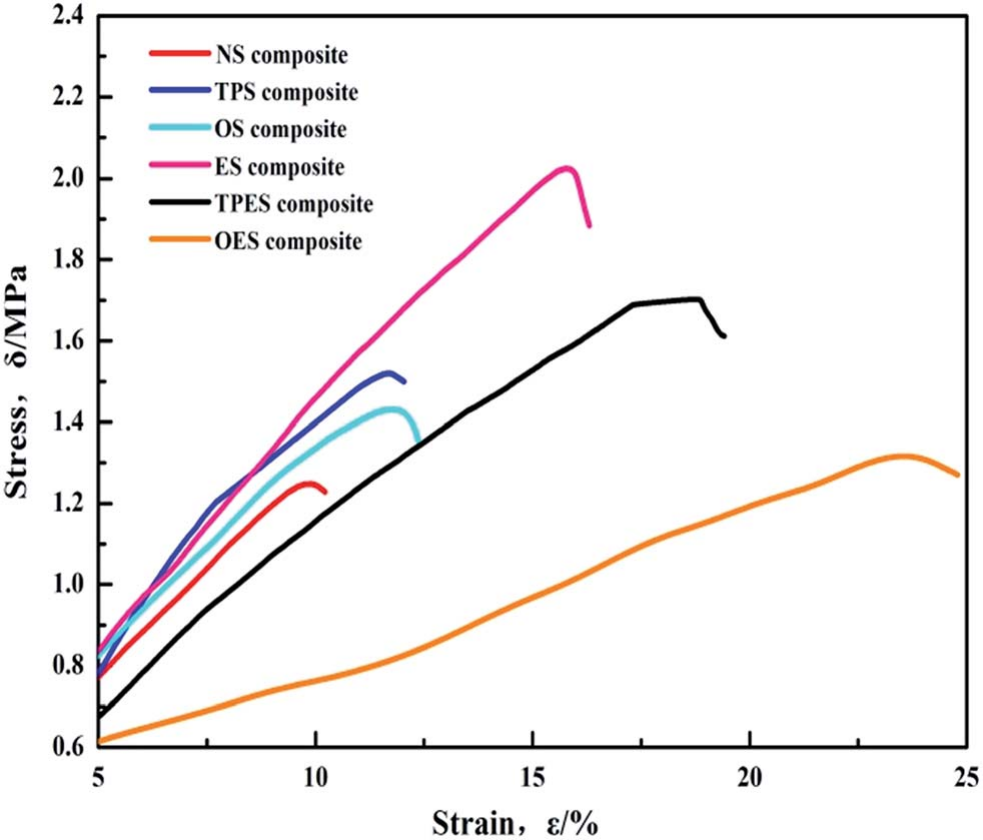
Stress–strain (*δ*–*ε*) curves of single-modified/cross-modified starch-based composites.

The tensile stress–strain test results of the NS-based composite (NS composite) and the single-modified/cross-modified starch-based composites are shown in Fig. 4. The results show that the tensile strength of the modified starch-based composites exceeds that of the NS composite (1.24 MPa). Compared with the NS composite, cross-modification starch-based composites require less deformation force but exhibit a larger deformation and smaller Young’s modulus. Single-modification starch-based composites require higher forces to deform, and exhibit larger deformations and higher Young’s moduli compared with the NS composite. Among the samples, the ES-based composite (ES composite) exhibited the largest tensile strength of 2.02 MPa. Compared with the NS composite, the ES composite showed a 61.6% and 55.3% increase in tensile strength and elongation at break, respectively. The Young’s modulus was almost the same as that of the NS composite. Despite the lower tensile strength compared with the ES composite, cross-modified starch-based composites exhibited better elongation at break and smaller Young’s moduli than single-modified starch-based composites. The OES composite exhibited the highest elongation at break of 24.8% and the minimum Young’s modulus of 5.57, showing a 136.1% and 54.3% increase compared with those of the NS composite, respectively. The tensile strength of the OES composite was 1.32 MPa. The specific results are shown in Table 1. In conclusion, single-modified starch-based composites exhibit high tensile strength but present poor toughness. Cross-modified starch-based composites exhibit low tensile strength but show good toughness.

**Table 1:** The results of the data from the tensile test

	NS	TPS	OS	ES	TPES	OES
Elongation at break (ε/%)	10.50	12.04	12.38	16.31	19.42	24.79
Young’s modulus (MPa)	12.18	13.53	11.91	12.57	9.24	5.57
Tensile strength (MPa)	1.25	1.52	1.43	2.02	1.70	1.32

**Fig. 5:**
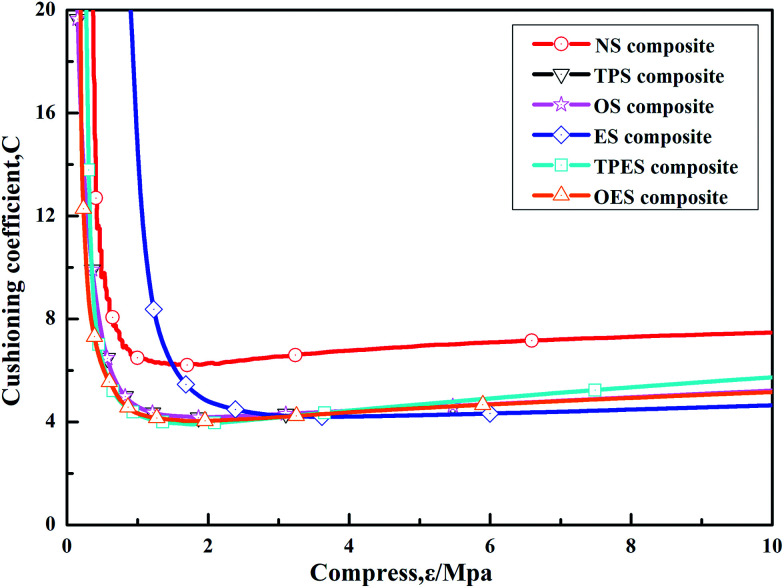
Cushioning coefficient–strain (*C*–*δ*) curves of single-modified/cross-modified starch-based composites.

The cushioning coefficient–strain test results of the NS composite and single-modified/cross-modified starch-based composites are shown in Fig. 5. The results indicated that the cushioning coefficients of single-modified starch-based composites and cross-modified starch-based composites were basically equivalent and were smaller than that of the NS composite. The values gradually decreased with the increasing applied force and then stabilized finally in the range of 4.8–5.2. The modified starch-based composites exhibited lower cushioning coefficients than the 7.5 of the NS composite, as well as superior cushioning performances. In conclusion, the single-modified/cross-modified starch can improve the cushioning properties of composites to a certain extent, but no difference was observed between the single-modified starch-based composites and the cross-modified starch-based composites.

After the process, the tensile strength of EPS was approximately 0.15 MPa, its compressive strength was approximately 0.3 MPa, and its corresponding minimum cushioning coefficient was 4 to 5.^41^ The minimum tensile strength (1.24 MPa) of the starch-based composites studied in this work was significantly superior to that of EPS. After starch modification, the cushioning coefficients (4.8–5.2) of the composites were similar to that of EPS. Thus, compared with EPS, starch-based composites exhibit obvious advantages in mechanical properties, which can become an interesting topic for future research.

### 3.2 Mechanism analysis of mechanical property changes in composites

To explore the microscopic mechanism of the single-modification/cross-modification of starch on the mechanical properties of the composites, the modified starches were analyzed by FTIR spectroscopy. In this study, FTIR spectroscopy was focused on the O–H stretching vibration absorption peak and the absorption peak was located at 3300–3600 cm^−1^. The formation of hydrogen bonds significantly changed the vibrational frequency of the hydroxyl groups. Strong hydrogen bonds indicated a low vibrational frequency of the hydroxyl groups and a wide vibrational band.^42^

**Fig. 6:**
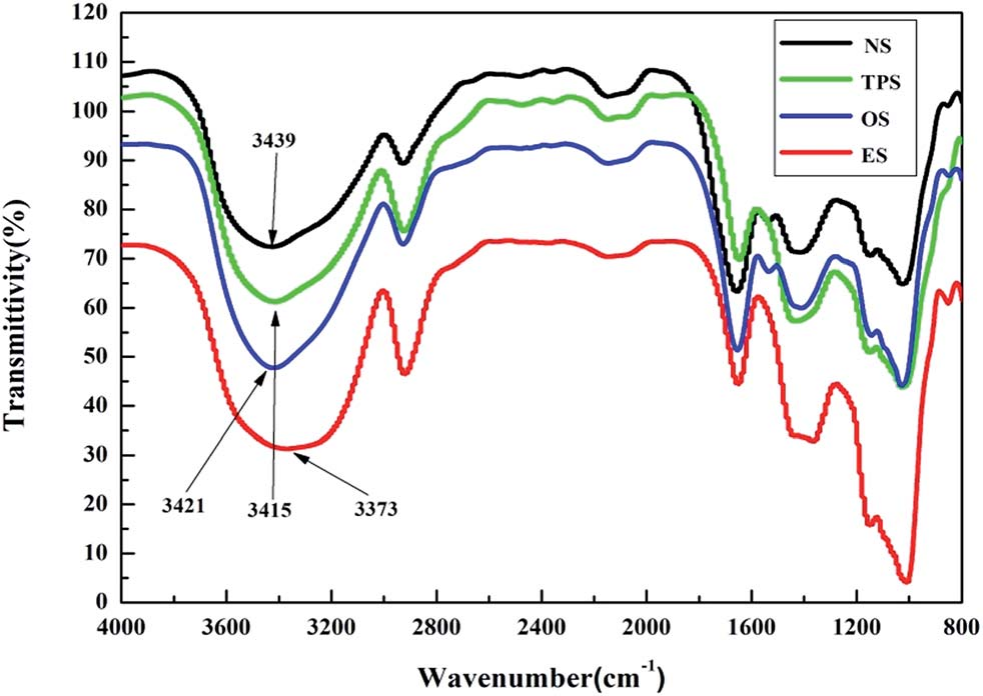
The infrared spectra of NS, TPS, OS and ES.

Fig. 6 shows the FTIR spectra of NS, TPS, OS, and ES. The results revealed that after single-modification of starch, strong hydrogen bonds were formed. Among the bonds, the hydrogen bond formed by the esterification modification was the strongest, whereas the hydrogen bond formed by the oxidation modification was weaker than that formed by the plasticization modification. This finding was in good agreement with the mechanical properties exhibited by the composites. This phenomenon showed that the internal structure of starch was destroyed by the modifier to a certain extent, resulting in strong hydrogen bonding. These conditions enabled the starch molecules to combine tightly with sisal fibers, and enhanced the tensile properties of the composites after single-modification. From a chemical point of view, electronegative ions such as O^2−^ can be easily accessible owing to the large number of hydroxyl groups in the starch molecule, which tends to combine with modifier binders to form highly polar H^+^ ions. Because of the high polarity of H^+^, it forms strong hydrogen bonds, demonstrating the accuracy of the infrared spectroscopy test. In this study, the ES composites with the best tensile strength, the TPES composite and the OES composite, are selected for analysis to study the difference between single-modification and cross-modification of starch in the formation of hydrogen bonds.

**Fig. 7:**
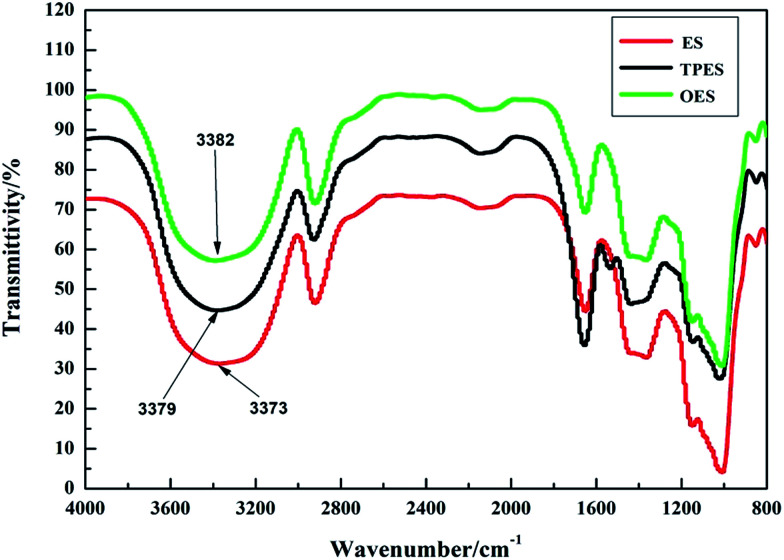
The infrared spectra of ES, TPES and OES.

Fig. 7 shows the infrared spectra of ES, TPES, and OES. The results showed that the hydrogen bonding formed by cross-modification was slightly weaker than that of ES due to mutual partial binding of modified agents. However, the tensile strength of cross-modified starch-based composites was significantly smaller than that of the ES composite. The phenomenon did not match the tensile strength (Table 1) because although the hydrogen bonds of the cross-modified starch-based composites were slightly weaker than those of the ES composite, the molecular structure of the starch was less damaged during esterification. The chemical bonds inside the ES were destroyed to a lesser extent, whereas the chemical bonds inside the OES composite were nearly destroyed. The chemical bonds inside the starch molecules and the hydrogen bonds of the ES composite were both undamaged. Thus, a large force is required for deformation, and ES samples are not easily deformed. However, cross-modification extensively damaged the molecular structure of starch, causing the chemical bonds to almost disappear during the stretching. In addition, only the hydrogen bonds played an important role. Less force was required during deformation and the easy deformation was due to hydrogen bonds being weaker than chemical bonds (we made this inference based on the cross-modified starch-based composites requiring less force to deform than the NS composite as shown in the ordinate of Fig. 4 and the fact that only chemical bonds played a significant role in the NS composite). Moreover, so many effective combinations were available. These combinations were well-knit in that the hydrogen bonds of cross-modified starch were extremely strong, with the tensile strength exceeding that of the NS composite; moreover, the ES composite was not easily snapped during stretching. However, the elongation at break was closely related to the degree of deformation, whereas the Young’s modulus reflected the ease of deformation as the slope of the curves show in Fig. 4. Increased deformation means a large elongation at break. In addition, the presence of easily-deformed composites indicates a small Young’s modulus. Thus, the cross-modified starch-based composites showed significantly lower tensile strengths, larger elongations at break, and smaller Young’s moduli than the ES composite.

**Fig. 8:**
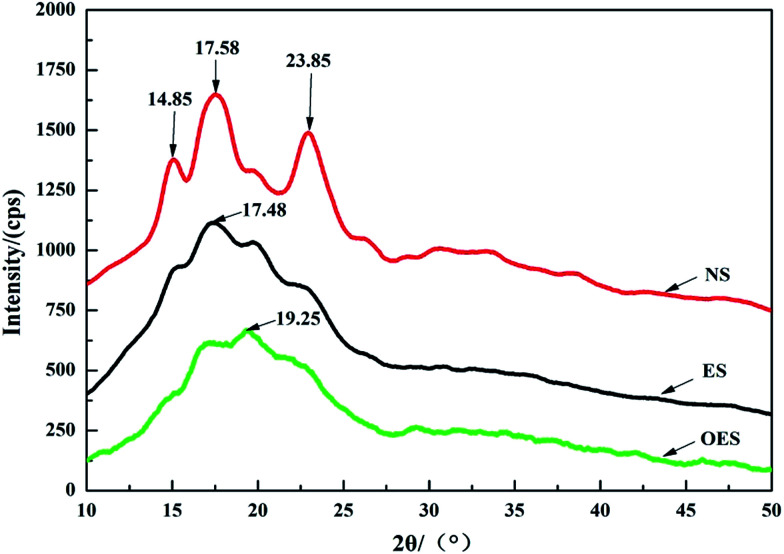
X-ray diffraction diagrams of NS, ES and OES.

In order to further study the effects of single-modification/cross-modification of starch on the mechanical properties of composites and the accuracy of FTIR spectroscopy, we selected the most representative modified starch (the single-modified starch was ES, and the cross-modified starch was OES) for XRD analysis. Fig. 8 shows the XRD spectra of NS, ES, and OES. In the analytical results, NS displayed a predominance of crystallinity type A and three sets of diffraction peaks at 2*θ* = 14.85°/17.58°/23.85°, correspondingly. ES presented one set of diffraction peaks located at 2*θ* = 17.48°. OES presented only one set of diffraction peaks located at 2*θ* = 19.25°. Furthermore, the figure shows that the crystalline types of NS, ES, and OES were significantly different.

Data were processed and fitted by MDI Jade software. The crystallinity index of NS was 19.68%, whereas the crystallinity index of ES was 14.58% and that of OES was 9.24%. The results indicated that the cross-modified starch exhibited a lower degree of crystallinity compared to the single-modified starch. The internal structure of the cross-modified starch was severely damaged. Numerous branches were exposed, enabling the starch to combine easily with sisal fibers. Thus, numerous good cell structures were formed, verifying the accuracy of FTIR spectroscopy. Tensile test results showed that the tensile strength of the OES composite was larger than that of the NS composite. Although the OES composite almost exclusively involved hydrogen bonds, and the NS composite only contained chemical bonds, numerous branches of cross-modified starch were exposed, allowing easy combination of these branches with sisal fibers. Therefore, many strong hydrogen bonds occurred in the composites, resulting in a large tensile strength compared to the NS composite, a large elongation at break, and a small Young’s modulus. This result proved the rationality of mechanical testing.

### 3.3 Internal structure analysis of starches and composites

**Fig. 9:**
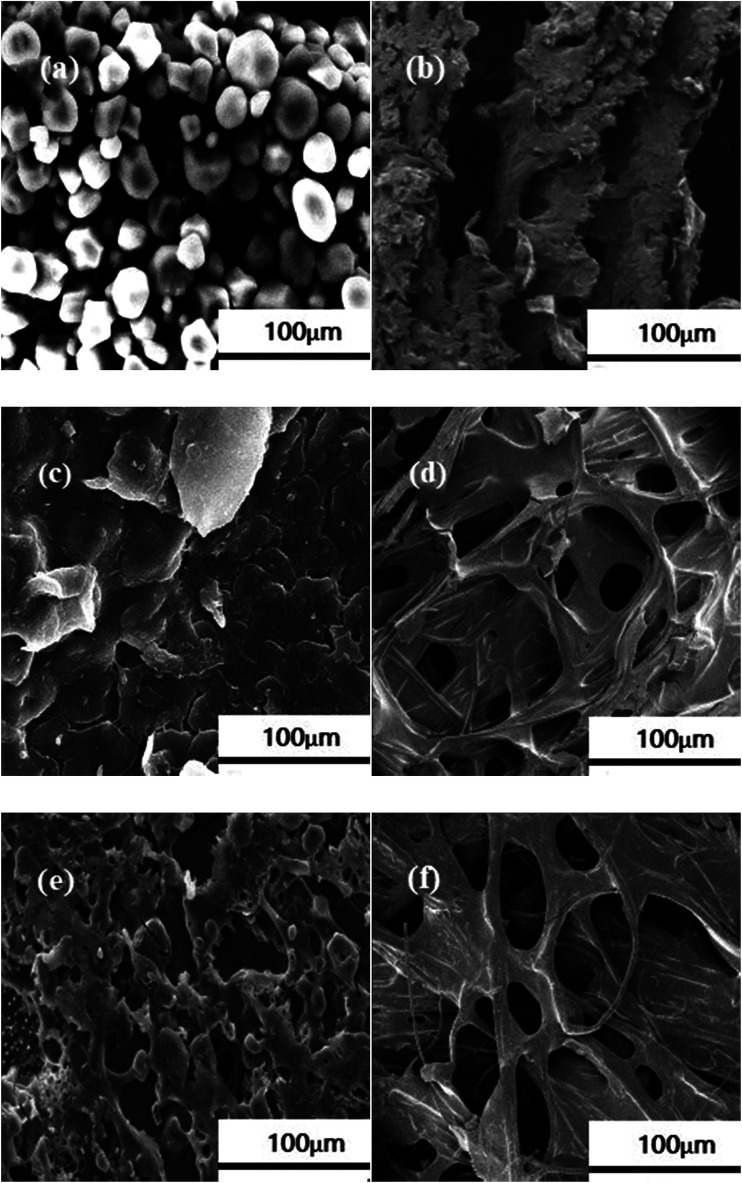
SEM images of (a) NS; (b) the NS composite; (c) ES; (d) the ES composite; (e) OES; and (f) the OES composite.

To further investigate the micro-mechanism of the composites, we conducted SEM analysis of NS, ES, and OES, and their corresponding composites, and the results are shown in Fig. 9. Fig. 9(a) shows that NS is tightly bound together by a series of colloidal molecules to form a relatively closed internal structure; thus, starch molecules are aggregated separately and are not compatible with sisal fibers in the NS composite. The composite also presented no cell structure inside (Fig. 9(b)). In consequence, these conditions lead to poor mechanical properties of the NS composite. After the esterification of starch, the destroyed starch still exhibited an evident structure (Fig. 9(c)). Starch molecules became closely connected with each other, leading to the fact that although the exposed branches of ES were combined with the fibers, the internal connection of the starch molecules showed a closed structure to a certain degree and most chemical bonds were retained, creating a few cell structures in the interior of the composite and an uneven distribution of the starch. The result is shown in Fig. 9(d). Fig. 9(e) shows that the molecular structure of OES was almost completely destroyed, and the starch molecules were relatively dispersed between the structures. Numerous branches were exposed, enabling the starch to easily combine with sisal fibers as shown in Fig. 9(f). According to the SEM analysis, the molecular structure of starch was minimally changed and most chemical bonds were retained after single-modification. The distribution of starch was not uniform, and the effective combination of starch and sisal fiber was low, resulting in a relatively small number of cells formed in the composites. However, after cross-modification of starch, the molecular structure was basically destroyed, and no chemical bonds existed, thereby exposing a large number of branches and allowing easy combination with sisal fibers. The starch matrix was evenly distributed in the sisal fiber surface, forming a large number of good cell structures. The accuracy of the XRD conclusions and mechanical test results was confirmed.

## 4. Conclusions

A new biomass-cushioned packaging product is composed of a starch-based composite with plant fiber as the skeleton and starch as the binder and is synthesized by a molding-foam method.

Cross-modification of starch improved the toughness of the composites, whereas single-modification of starch increased the tensile strength of the composites. The ES composite improved the tensile strength of the composite by 61.6%, whereas the OES composite improved the elongation at break and the Young’s modulus by 136.1% and 54.3% respectively, compared with the NS composite. The cushioning coefficient of the modified starch-based composites was significantly lower than that of the NS composite, and this result was basically the same as that of EPS. Starch-based composites offer evident advantages in mechanical properties compared with EPS. Starch-based composites can be the focus of future studies.

FTIR spectroscopy showed that starch formed additional strong hydrogen bonds after modification. Although the hydrogen bond strength of ES was slightly higher than that of the cross-modified starch, the tensile strength of ES was obviously stronger than that of the cross-modified starch because of the chemical bonds. The chemical bonds inside the starch molecules and the hydrogen bonds of the ES composite existed simultaneously, whereas only the hydrogen bonds played an important role in the OES composite. This is because cross-modification extensively damaged the molecular structure of starch, causing the chemical bonds to almost disappear for the OES composite. In addition, less force was required during deformation, and the easy deformation was due to hydrogen bonds being weaker than chemical bonds for the OES composite, compared with the ES composite. Specifically, the OES composite has a good toughness, whereas ES has a high tensile strength. The results of the XRD analysis showed that ES has a high crystallinity and a relatively intact internal structure; thus more chemical bonds still existed and fewer branches were exposed to combine with fibers compared with OES. The results of XRD were consistent with the analysis of the FTIR spectra.

The SEM images showed the evident structure of ES. ES presents few bare branches and a closed structure to a certain degree, resulting in low binding of starch and sisal fibers in the ES composite. The cell structure was poorly formed and sparse. Starches were unevenly distributed on the surfaces of fibers. However, the molecular structure of OES was basically completely destroyed, and the starch molecules were relatively dispersed. This phenomenon exposed a large number of branches and allowed easy combination with sisal fibers, forming several good cell structures. Starches were evenly distributed on the surfaces of sisal fibers. The results of SEM were consistent with the analytical results from XRD and FTIR spectroscopy.

## Conflicts of interest

There are no conflicts of interest to declare.

## Supplementary Material
